# Circadian succession of molecular processes in living tissues

**DOI:** 10.1186/s12920-018-0325-2

**Published:** 2018-02-13

**Authors:** Abeer Fadda, Mohammed El Anbari, Andrey Ptitsyn

**Affiliations:** 1 Sidra Medicine, PO Box 26999, Doha, Qatar; 2 Present Address: Gloucester Marine Genomics Institute, 2 Blackburn Center, Gloucester, MA 01930 USA

## Abstract

**Background:**

Oscillations of different origin, period and amplitude play an important role in the regulation of cellular processes. Most widely studied is the circadian or approximately daily variation in gene expression activity. Timing of gene expression is controlled by internal molecular clock keeping steady periodic expression. In this study, we shift attention towards a broad range of periodically expressed genes involved in multiple cellular functions which may or may not be under direct control of the intrinsic circadian clock. Are all molecular functions represented in expressed genes at all times? Alternatively, are different molecular functions performed at different times? Is there a pattern of succession for molecular processes and functions throughout their daily activity period?

**Results:**

To answer these questions, we re-analyzed a number of mouse circadian gene expression data available from public sources. These data represent the normal function of metabolically active peripheral tissues (white adipose tissue, brown adipose tissue, liver). We applied novel methods for the estimation of confidence in phase assignment to identify groups of synchronous genes peaking at the same time regardless of the amplitude or the absolute intensity of expression. Each synchronous group has been annotated to identify Gene Ontology (GO) terms and molecular pathways. Our analysis identified molecular functions specific to a particular time of the day in different tissues.

**Conclusion:**

Improved methodology for datamining allowed for the discovery of functions and biological pathways in groups of genes with synchronized peak expression time. In particular, such functions as oxidative phase of energy metabolism, DNA repair, mRNA processing, lipid biosynthesis and others are separated in time. This timewise compartmentalization is important for understanding the cellular circuitry and can be used to optimize the time of intervention with drug or genome medication.

**Electronic supplementary material:**

The online version of this article (10.1186/s12920-018-0325-2) contains supplementary material, which is available to authorized users.

## Background

A wealth of knowledge has been generated on the sequence of physiological and behavioral processes that oscillate within the diurnal cycle of a mammalian life. The triggers and downstream effectors of circadian rhythm are well characterized on an organismal and molecular level, and several tissue types have been assessed for periodicity of global gene expression. The question of how many genes oscillate in a circadian (i.e. approximately daily) period has been the subject of many debates [1–4] and the answers vary with tissue type, experimental conditions, technology, and statistics used to infer oscillating genes. With the exception of the well-studied clock genes, and while the identity and function of most of the oscillating genes is known, the significance of the timing of their oscillation has not been fully dissected. However, one study has produced an interesting observation of gene expression in connection with sleep cycles [5]. The report shows a succession of molecular functions among genes expressed at different times. The authors did not connect these observations with circadian molecular clock, but used the same diurnal time scale in their experiments. They point out that, in nervous tissue, sleep is an important adaptation to the cyclic nature of macromolecule biosynthesis. We may ask the next question: what happens in the other tissues? Does the diurnal rhythm of biosynthesis impose temporal limitations on the functioning of the other tissues in a way similar to sleep requirements for neurons?

In each of the tissues studied, researchers identified the molecular pathways and functions associated with the circadian genes and interpreted the results in the context of tissue type. For example, circadian genes in cardiomyocytes were found to be involved in transport, transcription, signal transduction, protein turnover, and metabolism [6]. In the liver, targets of the core clock genes were found to be involved in metabolic pathways in cancer and insulin signaling [7]. Zhang et al. tried to follow the peak times of certain pathways in different tissue and reported on some of them [4]. However, a global picture of the sequence of molecular functions oscillating through the diurnal cycle has not yet been outlined.

Based on the previous observations and these questions we can formulate the central hypothesis for this small study: if other tissues require certain stretches of time in their diurnal cycle to recharge and replenish like the nervous tissue does in a sleep cycle, then some GO terms and pathways must show significant association with certain times. Alternatively, the pathways and functional annotation terms may be distributed uniformly in time (i.e. spare genes peaking at different time, but the pathway they form is active at all times).

To resolve this dilemma we re-analyzed several datasets of time-course gene expression in different tissue in mice: liver tissue and white and brown adipose tissue data set previously described in Zvonic et al. [8]. We used a confidence interval to assess their periodicity within predefined time slots, and came up with sets of genes oscillating in sequential phases. We then interrogated the functions of the genes by doing a gene ontology enrichment analysis. We concluded that some molecular functions and cellular processes are indeed triggered at certain times of the day in a tissue-specific manner.

## Methods

**Fig. 1:**
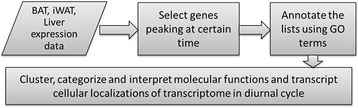
Overview of the analysis workflow

The overview of the analysis is given in Fig. 1. We used the same data as in a number of previous in methodology studies [9–12].

### Data processing

The data were normalized using a quantile algorithm similar to the one described by Bolstad et al. [13]: ${x}_{norm}={F}_2^{-1}\left(G(x)\right)$ where *F* is the distribution function of the actual sample, and *G* is the reference distribution function. In this case the reference distribution is estimated by an average of sample distribution for the entire data set. We used a seven-point Savitzky-Golay algorithm [14] for additional smoothing of the reference distribution. Each expression profile is scaled to its own standard deviation (i.e. z-scored) to achieve a uniform range for further testing and selection.

### Selection of oscillating expression profiles

In this study we did not try to assess how many genes are expressed in oscillating pattern. Low sampling rate renders all periodicity tests underpowered and the numbers of truly oscillating genes underestimated [1]. Earlier analysis of the same data reported numbers from roughly 20% [8] to over 90% [11] of genes with detectable baseline oscillation, depending on the algorithm applied. It is reasonable to assume that genes for which the time of peak expression can be identified with more certainty are also likely to be rhythmically expressions. The test for a certain peak time is applied to each profile independently. However, independence of oscillation pattern in gene expression could not be assumed. Living cells are known to have more than one oscillator [15], but these oscillators are normally synchronized to the rhythm of the circadian molecular clock, active in peripheral tissues. Testing individual expression profiles for periodicity in only one circadian frequency we are looking for manifestation of the same factor, hence not testing independent hypotheses. For these reasons FDR correction has not been applied to reduce the number of selected genes.

### Determining oscillatory phase and phase confidence estimation

We chose the phases of oscillation such that the peak of each phase coincides with sample harvest time, and for each phase we set a confidence interval around the peak time. The Bray data was assayed every 3 h so we have a total of 8 phases with a 6 h confidence interval for each phase. For example, phase 1 has a confidence interval between zeitgeber 21 and 3 h; phase 2 has an interval between 0 and 6 h, and so on. The Zvonic et al. data was assayed every 4 h, so we have a total of 6 phases with confidence intervals spanning 8 h. We estimated correlation to ideal cosine function (discrete, generated with the same number of time points as corresponding expression data) and classified all expression profiles to the nearest match. For the next step, we applied Maximum Entropy Bootstrap algorithm to estimate confidence level in our phase assignment. The algorithm description and R code are available in the supplemental materials (Additional file 1).

### Functional annotation

We used DAVID [16] for functional annotation of microarray probe sets as well as statistical enrichment of phase group by GO terms and Kyoto Encyclopedia of Genes and Genomes (KEGG) pathways. The complete annotated list is provided in the supplemental materials (Additional file 2). The data does not allow for the assumption of independent testing for highly dependent and overlapping set of pathways. Nevertheless, the standard set Bonferroni, Benjamini and FDR corrections of DAVID are provided for reference. Functional annotation clusters are presented in Additional file 2. Affymetrix probe sets were submitted as gene lists while the entire microarray was used as a background. Thomson-Reuters Metacore software was used in the early stages to perform first pilot analysis and to generate Tables 1, 2, and 3.

**Table 1:** Phase enrichment of iWAT data for molecular functions

Molecular functions	iWAT Zt0	iWAT Zt4	iWAT Zt8	iWAT Zt12	iWAT Zt16	iWAT Zt20
binding	0.15	0.09	**0.00**	**0.00**	**0.00**	**0.00**
enzyme regulator activity	0.74	0.88	0.74	0.25	0.17	**0.02**
protein binding transcription factor activity	0.53	0.70	0.25	**0.02**	**0.03**	0.95
structural molecule activity	0.60	**0.05**	**0.00**	**0.05**	0.98	0.28
Number of genes	26	42	214	125	111	98

Each table cell shows estimated *p*-value for significance of over-representation of a particular GO category (row) among genes peaking at certain time (column). Cells with *p*-values 0.05 and below are highlighted. The last line shows the absolute number of annotated genes peaking at particular time

**Table 2:** Phase enrichment of BAT data for molecular functions

Molecular functions	BAT Zt0	BAT Zt4	BAT Zt8	BAT Zt12	BAT Zt16	BAT Zt20
binding	**0.00**	**0.00**	**0.02**	0.07	**0.00**	**0.01**
catalytic activity	0.42	0.32	**0.00**	**0.00**	**0.05**	0.41
structural molecule activity	0.48	0.06	0.23	0.96	**0.01**	**0.05**
transporter activity	0.81	0.90	**0.05**	0.56	**0.02**	0.75
Number of genes	53	107	154	142	142	60

Each table cell shows estimated *p*-value for significance of over-representation of a particular GO category (row) among genes peaking at certain time (column). Cells with *p*-values 0.05 and below are highlighted. The last line shows the absolute number of annotated genes peaking at particular time

**Table 3:** Phase enrichment of liver data for molecular functions

Molecular functions	Liver Zt0	Liver Zt4	Liver Zt8	Liver Zt12	Liver Zt16	Liver Zt20
binding	**0.02**	**0.01**	**0.00**	**0.00**	0.12	**0.00**
catalytic activity	0.19	0.19	**0.02**	**0.00**	0.44	**0.03**
enzyme regulator activity	0.57	0.11	**0.03**	**0.04**	0.29	0.33
protein binding transcription factor activity	0.50	0.88	0.16	0.06	0.47	**0.02**
structural molecule activity	0.32	0.64	0.62	0.88	**0.01**	0.13
transporter activity	0.90	0.12	**0.02**	0.85	0.10	0.16
Number of genes	65	131	158	171	130	63

Each table cell shows estimated *p*-value for significance of over-representation of a particular GO category (row) among genes peaking at certain time (column). Cells with *p*-values 0.05 and below are highlighted. The last line shows the absolute number of annotated genes peaking at particular time

## Results

### Prevalence of circadian rhythm in baseline gene expression

Previous analysis of the same data [8] first reported over 18% of microarray probe sets oscillating in circadian rhythm [9] and then over 90% with development of new methods based on digital signal processing [11]. This previous observation led to conclusion that the non-oscillating fraction of transcripts is either small or non-existing [1]. Each probe set loosely corresponds to one transcript, thus one gene can be represented by one or a few probe sets. The oscillating probe sets include the entire range of expression. Some of the oscillating transcripts show high fidelity and synchronization with their known co-expressed genes, even though their abundance is too low to call present by conventional methods [17]. For this reason, we did not pre-select transcripts or probe sets by the level of expression. We used the entire data regardless of the level of expression so long as the signal to noise ratio was high enough for Pt-test as described in Ptitsyn et al. [10].

### Succession of prevailing GO terms in circadian cycle

**Fig. 2:**
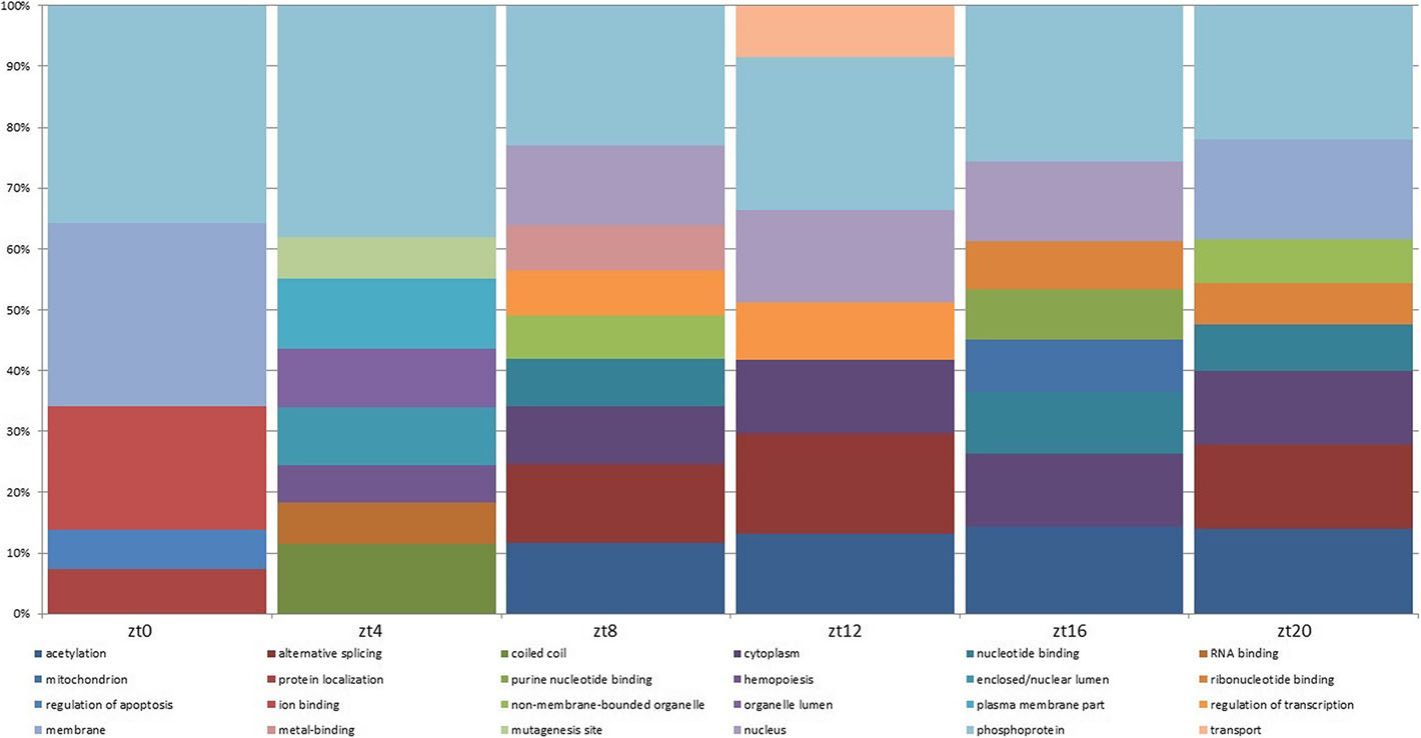
Succession of prevailing GO terms in circadian cycle. Only first ten most prevalent GO terms are shown for each phase group (column). The vertical axis shows relative abundance of a particular GO term. The complete table of GO annotation for each phase is available in supplemental materials

The subset of pronouncedly oscillating genes selected for further analysis in this study was the same as reported in the original analysis [8]. Application of phase confidence filter has significantly reduced the numbers of genes for further investigation. In IWAT, we selected 252, 47, 223, 128, 117 and 103 microarray probe sets peaking at zt0, zt4, zt8, zt12, zt16 and zt20 with high confidence (*p* < 0.05) correspondingly. These numbers are much less than the total numbers of oscillating genes and account only for the genes with peak activity at the time the sample is taken and not anywhere between sample collection time points. Functional annotation of the lists of genes with GO terms, PIR keywords and other features (available through DAVID [16]) reveals differences between phase groups. The overview of expression landscape is presented in Fig. 2. The complete list of genes and GO terms prevalent in transcriptome at different times of the day is given in the supplemental materials (Additional files 2, 3, and 4). The difference concerns both cellular localizations and molecular functions. Early phases are dominated by GO terms associated with the nucleus. Later, terms associated with cytoplasm, cellular membrane, and Golgi apparatus become more prevalent.

The patterns on Fig. 2 are formed by an abundance of annotation terms in fractions of genes peaking at a particular time (from zeitgeber+ 0 h at 4-h intervals). The color area is proportional to the number of genes annotated with corresponding terms. Annotation terms returned by DAVID are manually curated to remove redundancy. Some terms, like “phosphoprotein” are abundant throughout all phases. Others like “coiled coil” at zt + 4 h appear only once. Notably common is appearance of the same terms in a few adjacent phases, for example “regulation of transcription”, which is abundant among genes peaking at zt + 8 and zt + 12 h and subside after that. This observation is likely to reflect the processes that take more than one 4-h interval in time.

### Different tissues exhibit varying patterns of molecular processes synchronization

The pattern in Fig. 2 is characteristic of gene expression in white adipose tissue. As it was noted earlier [9], synchronization patterns are highly variable in different tissues. A more recent study of Zhang et al. [4] compared more different tissues and came to the same conclusion. The difference between tissues remains clear when we shift the focus from separate genes to large categories of annotation terms. Tables 1, 2, and 3 trace the occurrence of the same Molecular Functions in iWAT, BAT, and liver (the overview generated using Thomson-Reuters Metacore software). The statistical significance of enrichment of each synchronous group of genes is estimated by a *p*-value with *p* = 0.05 cutoff (highlighted cells). The total number of annotated genes peaking at certain times in these table is lower than the numbers of selected peaking probe sets due to probe redundancy and insufficient annotation of some probes. Nevertheless, the numbers are sufficient to see that different function dictates different synchronization pattern for each organ and tissue. Even though the same genes representing the same molecular functions might be active, two samples from different tissues are different at any given point of time. The overall pattern of gene synchronization is also different between tissues, even between white and brown fat. This difference is apparent even when very general GO categories are used, like those in Tables 1, 2, 3.

## Discussion

The facts that a larger part of the transcriptome experiences diurnal variations in baseline level of expression and groups of genes can be identified as peaking at the same time still leave at least two possibilities. First, genes peaking at the same time may represent different pathways and molecular processes; the functions on cellular level may not follow the same rhythm as separate genes. Second, genes peaking at the same time may reflect certain molecular and cellular processes; in this case synchronization of peak expression is functional and regulated. To test these alternative hypotheses, we have to identify the peak time of gene expression. This question is far from trivial when the data is sampled at extremely low rate, most typically once every four hours which provides only six time points per period. Genes that peak at a time between observation points may still appear periodic and pass the periodicity test. However, when characterizing commonalities among genes peaking at one time we need more than an assumption that this group of genes is synchronous. For this purpose, we employ a bootstrapping algorithm that allows selection of genes peaking at or near the time of observation (i.e. at one of the sample collection points spaced by four-our intervals) with 90% confidence (*p* < 0.1). This additional filtering leaves much fewer numbers of genes to characterize, but still sufficient for the pathways analysis. Confidence in selection of genes peaking within a certain time interval is an essential novelty in our methodology. It allows focusing on smaller groups of genes for which the true peak time can be estimated and produces a clearer pattern of molecular functions and pathways active at the stretch of time in vicinity of a specific sampling point.

In this study we intentionally keep only a course-grained view on molecular pathways. Functional annotation of genes peaking at a certain time in circadian cycle has been performed in multiple studies. The recent example of Zhang et al. attempts this kind of analysis on a larger scale scale [4]. However, the interpretation depends on the definitions of gene sets, pathways and other unions. Many pathways are found by tracing gene regulation and protein interaction around known disease-related genes and include multiple cellular processes with no regard of the time. On the other hand, most general annotation terms like subcellular localization or metabolic reactions can be found as parts of multiple pathways. Following the example of Mackievicz et al., we try to identify most general annotation terms associated with genes peaking at a certain time, similar to macromolecule synthesis genes associated with sleep function in nervous tissue.

In the Mackievicz et al. experiment, the researchers compare expression patterns between undisturbed control and a sleep-deprived cohort in the same timeline. One possible interpretation of these results can be based on assumption that sleep deprivation artificially halts the natural succession of molecular processes. Sleep-deprived animals have a large portion of cellular processes in their brain cells halted while the same processes in control group follow the normal diurnal cycle. Then, we can hypothesize that comparing gene expression between halted by sleep-deprivation and unrestricted groups has similarities to the comparison of expression patterns in the same group, but across the phases of sleep-awakening cycle. In the Mackievicz et al. experiment, the authors did not investigate differential expression between time points in the unrestricted control cohort. The data we analyze in this study was acquired through the normal diurnal cycle. Mice were awakened before sacrificed, but sample collection was finished in minutes and it is highly unlikely that the pattern of relative mRNA abundance has been significantly disturbed. Therefore, in the Zvonic et al. experiment, examining the lists of genes peaking at different time points should produce results similar to the lists of differentially expressed genes in the Mackievicz et al. experiment. Indeed, in both experiments we can clearly observe clusters of GO and other annotation terms in succession over time.

In Mackievicz et al., the authors argue that sleep is a special adaptation that allows animals with highly developed nervous system perform the essential macromolecule synthesis and complete the diurnal succession of cellular functions. Some of those functions cannot sustain normal brain activity, which manifests in a radical change of behavior. If these conclusions are correct, other tissues also go through the same cycle of cellular housekeeping. However, the tissues observed in our study do not modulate the animal behavior as much as the central nervous system does. Nevertheless, our observations give a reason to believe that the principal function of all tissues (or at least those we looked at) is also disrupted or reduced at certain times of the day.

**Fig. 3:**
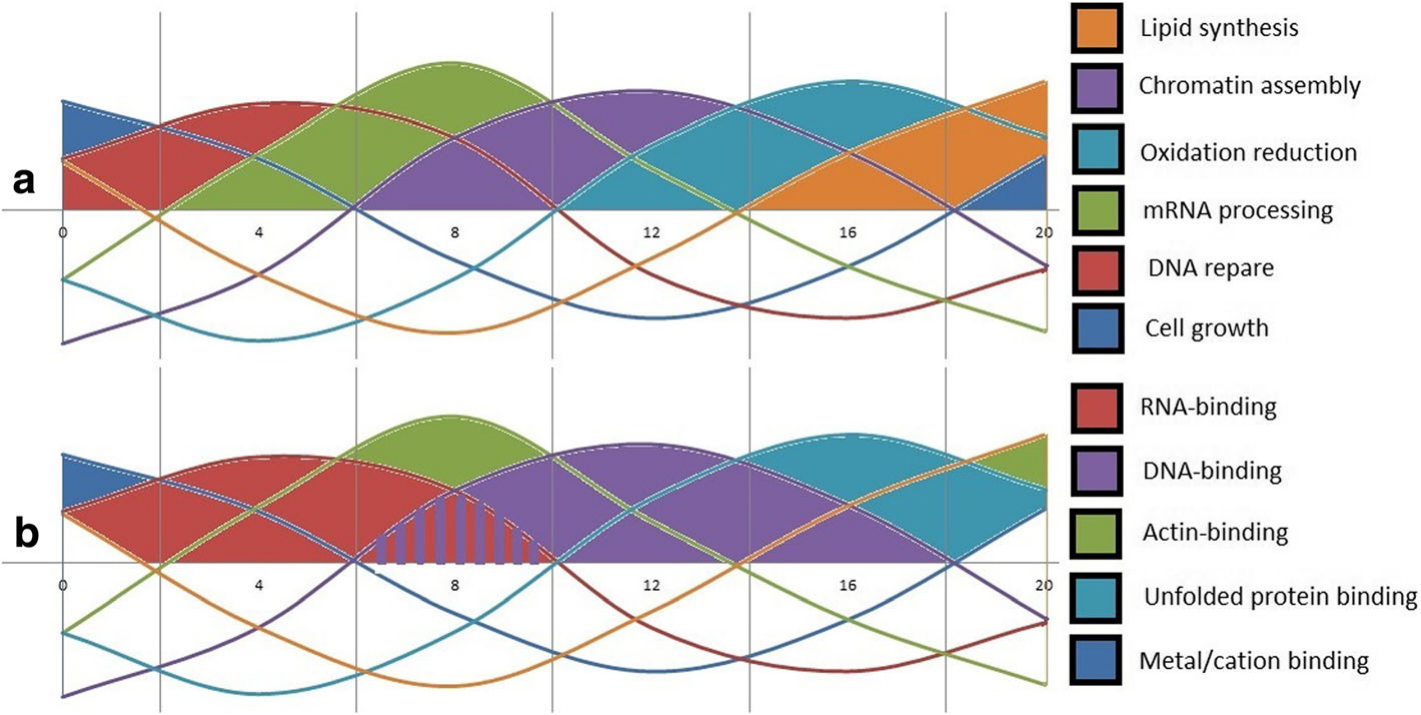
Peaking of time-specific gene functions in murine white adipose tissue. Succession of Biological Processes (**a**) and Molecular Functions (**b**) peaking at specific time of diurnal cycle. The diagram Expression of DNA-binding genes follows expression of RNA-binding genes with an overlap while acting-binding genes are peak twice a day

Fig. 3 shows selected examples of functional categories peaking that can be attributed to a certain time only or cover more than one period in succession. Each curve is a line plot of the average values for the genes peaking at certain four-hour intervals (marked on the horizontal axis). Overall the pattern is consistent with canonical succession of processes in cell biology. The wave of RNA-binding genes is succeeded by a wave of DNA-binding and chromatin assembly genes. Actin-binding genes peak twice, which probably reflects the peaks of transporting activity for different macromolecule components of cellular machinery. Peak of expression for gene involved in unfolded-protein utilization coincides with the peak of redox genes and follows the chromatin assembly. Remarkably, lipid synthesis in white fat is marked by gene activity in a late phase (zt + 20 h) right after the peak of redox genes.

The patterns we observe reflect only gene expression activity and not the entire tissue physiology. A pool of already synthesized and deployed protein may function throughout the times when genes responsible for particular function are inactive. However, the capacity to accommodate higher activity or respond to a signal is limited to the pool of available proteins while activation of transcription takes time and energy.

## Conclusions

Based on our computational observation, we can theorize that irregularities in life (such as an unusually large or untimely meal) may shift the balance from redox to lipid synthesis or from lipid synthesis to cell growth based on the state of preparedness of a cell to a particular function at specific time in a diurnal cycle. However, this and other theoretical concepts will benefit from experimental corroboration.

## Additional files

Additional file 1: R code for the analysis of confidence in phase assignment. (PDF 66 kb)

Additional file 2: Gene Ontology Charts. (XLSX 224 kb)

Additional file 3: Gene Ontology Clusters. (XLSX 298 kb)

Additional file 4: Gene Ontology Localizations. (XLSX 167 kb)
